# Training and HIV-Treatment Scale-Up: Establishing an Implementation Research Agenda

**DOI:** 10.1371/journal.pmed.0030304

**Published:** 2006-07-04

**Authors:** Elizabeth A McCarthy, Megan E O'Brien, William R Rodriguez

## Abstract

McCarthy and colleagues discuss the various approaches to training the health workforce for an expanding HIV treatment program in a resource-limited setting.

The provision of HIV treatment and care in resource-limited settings is expanding rapidly. Health-worker training is one of many factors critical to the rapid scale-up of high-quality care [ 1–6]. Large numbers of health workers require HIV training; yet, few countries have a comprehensive training plan, a clear assessment of ongoing training needs, a plan to operationalize training on a large scale, or adequate funds budgeted for training. In this setting, an extensive variety of HIV-related training programs have sprung up over the past few years. Unfortunately, there are limited data measuring their effectiveness, and there is no consensus about what constitutes effective training.

Underlying the looming challenge in health-worker training, most resource-limited countries face a chronic shortage of trained health-care providers; chronic understaffing impedes the ability to adequately train health workers in HIV care. First, removing clinicians and nurses from active clinics for training purposes intensifies the strain on clinical care systems. Second, professional programs for physicians and other health workers are commonly lacking. For example, several countries in Africa and the Caribbean—including Botswana, Lesotho, and the Bahamas—do not have medical schools, and must send students outside of the country for basic professional training (see http://imed.ecfmg.org/main.asp; Table 1). Finally, trained workers (and potential recruits) commonly leave the public health sector for better compensation, benefits, working conditions, and job satisfaction found in other sectors and other countries—the “brain drain” phenomenon—further exacerbating the human resource crisis [ 7–12].

**Table 1:**
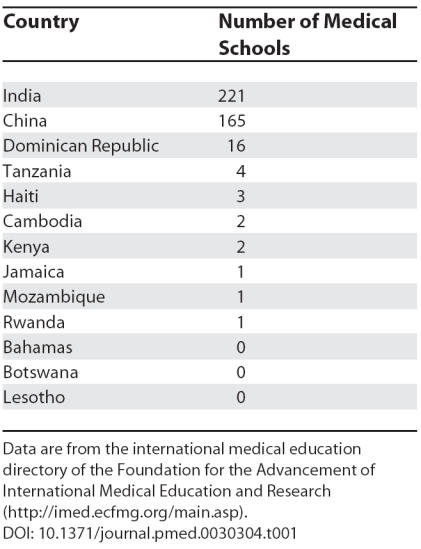
Medical Schools in Selected Countries

Faced with these challenges, and with the rapid pace of HIV-treatment expansion, few resource-limited countries have sufficient internal resources to address their training needs. As a result, most countries have collaborated with external partners to develop health-care-worker training programs, and/or to bring in expatriate specialists to provide training, at least in the initial phase of scale-up. Often, these training efforts are poorly coordinated with national training priorities, lack evidence to support their effectiveness, and are driven largely by foreign partners. As a result, many training redundancies exist alongside large, unmet training needs.

We gathered information on global HIV training through a thorough review of the published peer-reviewed literature, internet sites, program reports related to training for HIV treatment in resource-limited countries, a survey of HIV training efforts in high-burden countries, and discussions with appropriate professionals in selected countries. Here, we review challenges and approaches to clinical HIV training, and suggest an agenda for implementation research—defined here as research into how proven interventions can be implemented to accelerate high-quality HIV-treatment scale-up—to address the question: what is the optimal approach to training the health workforce for an expanding HIV-treatment program in a resource-limited setting?

## Training Appropriate to the Model of Care

The design of a national health-worker training program to support the expansion of quality HIV treatment should be tightly linked to the way in which HIV care and treatment are delivered in the respective country. Many national programs, such as those of the Bahamas [ 4], Botswana [ 5], and Uganda [ 13], initiated HIV treatment by following a “vertical” specialty HIV-clinic model in which the majority of HIV treatment is provided by HIV specialists. Training according to this approach targets the creation of multidisciplinary HIV-care teams who provide care predominantly, or exclusively, for patients with HIV.

At the other end of the spectrum is the public health model of care delivery [ 14, 15], where HIV care and treatment are provided by primary health-care providers who are trained in basic aspects of HIV care for adults and children and to recognize conditions that warrant referral to a specialized setting [ 16]. Training in advanced aspects of HIV care is reserved for a small cadre of specialists. A hybrid of these two models occurs when a national program starts its treatment program in the specialty model, but decentralizes HIV services to peripheral facilities. In this case, HIV care and treatment may be provided in a primary health-care setting by primary health clinicians, or by an HIV-care specialist.

In the vertical model, training in HIV care relies on a highly centralized training program driven by a small group of expert trainers, with a core curriculum that can be quickly and easily updated to keep pace with changes to practice and guidelines, and short intensive trainings for small groups of trainees. Parallel systems are often established for training in laboratory methods, counseling and patient education, data collection, and pharmacy and supply management. As programs decentralize into a public health model, training decentralizes accordingly. Short, intensive trainings in a central setting become less practical. The cadre of trainers and curricula must be expanded, and systems must be implemented to allow for curricula review, updates, and distribution of continuing medical education (CME) materials.

## Training Decisions amidst a Crisis in Human Resources for Health

The human-resources-for-health crisis in resource-limited countries is a substantial obstacle to scaling up HIV-treatment programs and is directly relevant to health-workforce training. Neither Mozambique, Rwanda, nor Tanzania, for example, has more than five physicians, 42 nurses, or three pharmacists for every 100,000 people ( Table 2) [ 17]. The United States, by comparison, has a density of 256 physicians, 937 nurses, and 88 pharmacists for every 100,000 people [ 17]. Chen et al. have linked low national staffing ratios to poorer health outcomes [ 18], and it is likely that this link extends to HIV care.

**Table 2:**
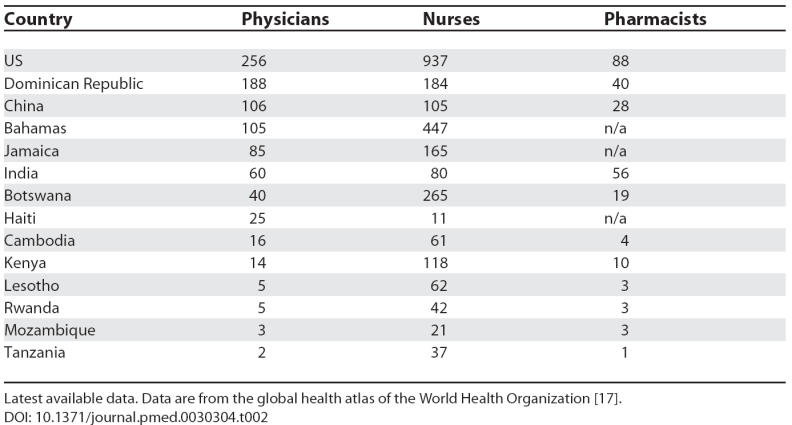
Human Resources per 100,000 People in Selected Countries

The HIV health workforce includes doctors, clinical officers, nurses, pharmacists, laboratory technicians, phlebotomists, counselors, program managers, data clerks, ancillary staff, and community health workers. The function of each category of health worker depends on the local model of care delivery, and is influenced by tradition, legislation, and local regulations. Variation in health-care-worker roles can be an obstacle to adapting generalized training tools and curricula to a specific setting. A recent study in the US found that nurse practitioners and physician assistants who specialize in HIV care provide better care than non-HIV expert physicians and comparable care to HIV-specialist physicians—a finding which could support the expanded role of nurses and clinical officers in HIV treatment in resource-limited settings [ 19].

As noted, many countries lack adequate pre-service training institutions for health-care workers, and must send clinicians outside of the country for professional training (see http://imed.ecfmg.org/main.asp). Postgraduate migration to other countries is common, exacerbating the human resource crisis [ 7]. In a setting where the need for health workers outweighs the number of health workers who are available, it can be difficult to entice workers to staff underserved areas, such as rural sites.

## Advantages and Disadvantages of Training Methodologies

There is scant evidence to support the effectiveness of one training methodology over another. Below, we present some of the advantages and disadvantages of the predominant training methodologies that have emerged ad hoc in the past few years. In Box 1, we briefly describe aspects of the training programs in Botswana, China, and the Dominican Republic.

### Pre-service education

Adding or enhancing relevant coursework during pre-service education for health professionals (e.g., medical schools, nursing schools) takes advantage of pre-existing programs without taking professionals away from the workplace as trainers or trainees. It helps address health-workforce needs, and ensures an adequate skill set among graduating professionals. However, it does not address the needs of those who have already completed their professional education, nor does it provide immediate solutions to urgent needs.

### Didactic training

Most training programs have emphasized centralized didactic training as the core training method. Didactic training, delivered as lectures in a classroom setting, is often used to convey large amounts of information at one time. It is often delivered in a centralized location, typically lasts a week or two, and can accommodate large numbers of trainees—requiring fewer trainers and resources than other methods, and allowing for standardization of the training's content. The classroom style is a familiar approach for many trainees, yet the translation of classroom knowledge to clinical practice can be challenging, especially if the curriculum is divorced from practical circumstances facing trainees. Trainees may not retain knowledge if it is not immediately applied to clinical practice. And, like all methods that take trainees and trainers away from their workplace, didactic training can temporarily exacerbate the strain on clinical care.

### Training of trainers

A training-of-trainers methodology is generally implemented when programs wish to provide didactic training at decentralized sites. Groups of health professionals are trained as “experts,” and expected to lead or facilitate future trainings. This approach attempts to expand the pool of trainers and leverage resources to build training capacity. An important downside of this method is the potential distillation of information as trainers get further removed from the original trainer's expertise and information, which can impact the quality of training and the resulting clinical outcomes. Some trainers may require training in educational methods and pedagogy beyond training in the management of HIV infection.

### Refresher course

It can be difficult for trainees with limited experience to absorb information from a didactic training. Trainees often benefit from practical experience at their own sites, followed by a refresher course. This affords trainees an opportunity to develop skills, before returning for ongoing training, that may add complexity and build on their classroom and practical experiences. It gives programs an opportunity to provide trainees with updated information, and affords trainees the opportunity to problem solve with each other.

### Distance learning

Using computer-based or video-based technology is another way to train health-care workers in resource-limited settings. This approach allows trainees to remain at their workplace, and has the added advantage of reaching a wide, geographically disparate audience with simulated cases that allow providers to test their knowledge without negative consequences to patients. These courses are inherently technology- and resource-intensive and require a certain degree of comfort with technological applications, but they reduce the need for trainers and allow trainees to move at their own pace.

While some programs, such as those in Botswana [ 5], Tanzania (S. Cress, Tanzania Country Director of the Clinton Foundation HIV/AIDS Initiative, personal communication), and the Dominican Republic (T. Brewer, Columbia University, personal communication) send multidisciplinary teams of health workers to didactic trainings, others focus didactic training exclusively on one discipline—such as physician trainings in Mozambique (G. Jagoe, Mozambique Country Director of the Clinton Foundation HIV/AIDS Initiative, personal communication). Often, workers from one clinic who attend the training are expected to bring back the information to their clinic and train the remaining staff, although this is rarely operationalized.

### Off-site clerkships (“attachments”)

Some programs, such as those in Botswana [ 5] and Kenya [ 6], complement didactic courses with opportunities to shadow experienced providers. During these off-site clerkships or “attachments,” trainees spend a block of time with a mentor at the mentor's clinical facility, which, ideally, is similar to their home clinic. Trainees gradually assume clinical responsibilities under supervision. The mentored environment allows the trainee to practice a skill with the comfort of having an experienced mentor to address questions and difficulties, and reinforces information provided during the didactic course. If the caseload and experience of the mentor are inadequate or if the attachment site differs significantly from the practice sites of the trainees, this type of training may be less relevant. Finally, attachments can take trainees away from their jobs for an extended period of time.

### On-site mentoring (“preceptorship”)

Botswana and Lesotho are both implementing national on-site mentoring, or “preceptorship,” programs that send experienced HIV-treatment professionals (nationals and/or expatriate health professionals) to sites of less-experienced providers for an extended period of time (several days to several months) to offer on-site mentoring. In Botswana, the preceptorship program builds on didactic training and an attachment at one of the four initial national treatment sites [ 5], while in Lesotho, the preceptorship program complements the decentralized trainings at antiretroviral treatment sites using the World Health Organization's Integrated Management of Adult and Adolescent Illness materials [ 16], which are tailored to the public health model of care delivery (J. Sun, Lesotho Deputy Country Director of the Clinton Foundation HIV/AIDS Initiative, personal communication).

The preceptorship training methodology has the same advantages as attachments, and also offers training specifically tailored for the trainee's work situation. Preceptorships can be particularly time-, labor-, and resource-intensive, and can require a large number of skilled mentors. While expatriate mentors are not always knowledgeable about local conditions, language, or policy, and may need to be licensed and/or registered to work as mentors, national mentors are in very short supply because of the human resource crisis and the fledgling nature of treatment programs in resource-limited countries. Most countries currently rely heavily on expatriate preceptors.

### Consultation

Some programs have developed a consultation system that allows newly trained providers to ask questions of experienced providers through direct phone calls, E-mail, call centers, or frequent site visits by the mentor. Consultation systems provide a support network that builds the confidence of newly trained providers. In Uganda, for example, the AIDS Treatment Information Centre hosts a call-in center that responds to providers' treatment questions (see http://www.w1.co.ug/atic-africa/www/about.php). Similarly, the Prince Leopold Institute of Tropical Medicine has developed an internet-based program, TELEmedicine, to enable their experienced providers to respond via E-mail to inquiries made by clinicians in resource-limited settings (see http://telemedicine.itg.be/telemedicine/Site/Default.asp?L=E&RND=2315701). One drawback to a phone or E-mail system of consultation is its reliance on communication technology.

### Case conferences

Another way to train providers is through case conferences: regular meetings to discuss complex problems in HIV care and to provide updates on practices or guidelines. Case conferences encourage a team approach to HIV care, help establish a network of HIV-care providers for informal consultation and/or referrals, and can reach a wide audience, especially with advanced internet-based conferencing software, where it is available.

### Twinning

An established relationship between two institutions to share expertise, which can be North–South or South–South, is referred to as twinning. One example of this approach is the collaboration between the Moi University Faculty of Health Sciences in Kenya and both the Indiana University School of Medicine and the Brown University School of Medicine [ 6]. Twinning increases resources for individual in-country institutions by facilitating a flow of funds and an exchange of information and expertise from one institution to the other. There is, however, a limit to the number of available twinning programs, and trainers from foreign institutions are not always knowledgeable about local conditions, language, or policy.

### Certification

Official recognition, or certification, of some degree of HIV-treatment expertise for trainees who complete a training program can be an incentive for completion. If accompanied by testing, it can ensure a minimum level of knowledge, and can be used to evaluate the effectiveness of the training. Re-certification can be the basis for a CME program. However, in addition to the potential for certification to increase bureaucracy and administrative costs, certification may be used by trainees to seek more lucrative positions outside of the country or with other organizations within the country.

### CME programs

These programs exist in countries with robust medical associations. Pre-existing CME systems can be used as a vehicle for HIV training, but are generally used to supplement a pre-existing knowledge base, not to train inexperienced providers.

## Establishing a National Training Plan

More data on the effectiveness and program costs of training are needed to help planners determine which options are optimal given a program's unique circumstances, including the size of the population requiring treatment, the care delivery model, the extent of local expertise, the existing public health infrastructure, health-care worker/population ratios, political will, nongovernmental organization involvement, and resources. Training programs would also benefit greatly from accurate forecasts of the demand for health workers [ 4]: the number of necessary staff needed immediately, the number of staff needed over time as programs scale up treatment, site locations, and the optimal number and mix of staff at each site. Such forecasts allow planners to determine the extent to which an investment in hiring and training additional health workers would affect HIV care and the extent to which it is critical to budget and resource-allocation decisions.

Training plans should anticipate common experiences—such as the permanent loss of trained health workers who take more lucrative jobs or burn out, the temporary loss of health workers who take leave or attend trainings, and worker illness and death from HIV infection. Some programs have chosen to train two individuals for every position, assigning each to spend half of their time at the HIV clinic and half elsewhere in the hospital or clinic. This reduces reliance on one individual, allowing each to miss clinical time without significant disruption. Without a buffer system to replace trained individuals, or the flexibility to train additional staff quickly, unexpected staff shortages create bottlenecks in clinic operation, slowing down the flow of patients and straining other staff. A forward-thinking national training plan will not only anticipate job loss but will also incorporate ways to avoid it. Approaches might include augmented salary, recruitment of staff to work in their home districts, improved staff-to-patient ratio, and adequate supplies.

Other considerations to be addressed when designing a training plan include the site of training, target audience, and content material. Training plans need to consider the optimal components of a training site, e.g., proximity to a health-care facility and an environment similar to what the trainee will experience at their home clinic. Training plans need to assess training previously received by workers, and decide whether to train one health cadre at a time or to train multidisciplinary teams together. Content material should match the reality on the ground, reflect local practice, and account for availability of drugs and diagnostic capabilities.

## Toward a More Evidence-Based Training Program

Decisions as to how best to train the health workforce in resource-limited countries are being made with limited data to support them. Few programs measure the impact of training on clinical outcomes. We have identified critical questions in Box 2 that correspond to key topics: model of care, human resources, and training delivery. We recommend that an implementation research agenda be established to address these questions.

## Conclusions

Training a robust health workforce is critical for sustainable HIV-treatment programs. The care delivery model, the roles played by different health workers, the number of workers needing training, resources available for training, and the phase of program development all significantly affect training design. Evidence to support these decisions must come from implementation research to answer the overarching question: what is the optimal approach to training the health workforce for an expanding HIV-treatment program in a resource-limited setting?

**Box 1. HIV-Related Training Programs in Selected Countries**

**Botswana**

The Knowledge, Innovation, and Training Shall Overcome AIDS (KITSO) AIDS Training Program, developed to support Masa, Botswana's national antiretroviral program, involves a combination of didactic training for multidisciplinary teams of core staff and on-site preceptorships with ongoing support for medical officers. The program was developed by the Botswana Ministry of Health, the Harvard AIDS Institute, and the Botswana–Harvard AIDS Institute Partnership, and has received support from the African Comprehensive HIV/AIDS Partnerships—a collaboration between the Government of Botswana, the Bill and Melinda Gates Foundation, and The Merck Company Foundation/Merck & Co., Inc. [ 5]

**China**

China focuses its training program on a short-term five-day training and a two-month in-service training. Most of the training is delivered by national experts from the HIV/AIDS Clinical Taskforce. The short-term training consists mostly of didactic lectures and case studies, while the in-service training emphasizes provider shadowing at an urban hospital. Additionally, in-service training geared toward county-level physicians is delivered at the Rural AIDS Clinical Training Center established in Anhui province [ 20].

**Dominican Republic**

The Dominican Republic uses a combination of methodologies for its training program. Most clinician training includes didactic sessions for multidisciplinary teams of key staff, followed by a short attachment for key staff and then by short on-site clinical mentoring visits by experienced local and expatriate clinical mentors (T. Brewer, Columbia University, personal communication).

**Box 2. Implementation Research**

**Model of Care**

- What are the advantages and disadvantages of parallel systems of specialty HIV clinics and primary health-care clinics?
- Does the most efficient model of HIV care delivery vary from urban to rural site?
- What is the optimal way to integrate HIV and other services (such as tuberculosis care, maternal and child health care, and HIV testing) to maximize patient capacity while minimizing resource needs?
- What is the impact on quality of care and cost if stable patients are managed by trained nurses, clinical officers, or generalist physicians instead of HIV-specialist physicians?
- What is the impact on quality of care and program scale-up rates if children with HIV are managed by doctors other than pediatricians?

**Human Resources**

- What is the optimal role for each health worker (i.e., physician, nurse, clinical officer) to maximize the patient capacity given fixed resources?
- How many health workers of each category are needed for a program to scale-up efficiently?
- Can existing health systems subsume HIV care and treatment without adding staff?

**Training Delivery**

- What is the ideal combination of training methodologies (didactic training, clerkship, on-site mentoring, on-going consultation, internet-based courses, etc.) to prepare providers to offer HIV care and treatment services?
- What are the most effective ways to reinforce knowledge and skills gained in training (e.g., CME, refresher courses, consultation)?
- Does inclusion of other causes of morbidity in HIV training, such as diabetes, tuberculosis, sexually transmitted infections, malaria, and other locally prevalent infections, lead to improved patient outcomes?
- Does inclusion of nutrition in HIV training lead to improved patient outcomes?
- What are the advantages and disadvantages of relying on local, national, or foreign HIV experts to provide HIV training?

## Abbreviation

CME: continuing medical education
